# Cardiac Findings Following Cerebrovascular Disease

**DOI:** 10.1161/JAHA.124.034131

**Published:** 2024-08-27

**Authors:** Marco H. Rohner, Catherine Gebhard, Andreas Luft, Martin Hänsel, Susanne Wegener

**Affiliations:** ^1^ Department of Neurology University Hospital Zurich and University of Zurich Zurich Switzerland; ^2^ Department of Cardiology Inselspital Bern University Hospital Bern Switzerland; ^3^ Cereneo, Center for Neurology and Rehabilitation Vitznau Switzerland

**Keywords:** acute ischemic stroke, cerebrovascular disease, hemorrhagic stroke, neurocardiology, transient ischemic attack, Ischemic Stroke, Transient Ischemic Attack (TIA), Cerebrovascular Disease/Stroke, Secondary Prevention, Intracranial Hemorrhage

## Abstract

**Background:**

Accumulating evidence suggests that cardiac findings after stroke are an important, yet understudied, manifestation of brain–heart interactions. Our aim was to investigate and compare cardiac findings after different cerebrovascular events (acute ischemic stroke, transient ischemic attack, and hemorrhagic stroke).

**Methods and Results:**

There were 7113 patients screened who were treated between December 2013 and December 2020 at the University Hospital Zurich for ischemic stroke, transient ischemic attack, and hemorrhagic stroke. Seven hundred twenty‐one patients without evidence of previous cardiac disease or presumed cardioembolic origin of their cerebrovascular disease and with at least 1 cardiac checkup were included. Clinical reports from the year following disease onset were screened for new cardiac findings, which were categorized as arrhythmia/electrocardiographic changes, myocardial alterations, valvular abnormalities, and coronary perfusion insufficiency. Differences in proportions of findings among groups were analyzed using the Pearson χ^2^ test or Fisher exact test. ECG changes were observed in 81.7% (n=474) of patients with ischemic stroke, 71.4% (n=70) of patients with transient ischemic attack, and 55.8% (n=24) of patients with hemorrhagic stroke (*P*<0.001). Myocardial alterations occurred often in all 3 groups (60.9% ischemic stroke [n=353], 59.2% transient ischemic attack [n=58], 44.2% hemorrhagic stroke [n=19]; *P*=0.396).

**Conclusions:**

Cardiac findings are frequent in patients with cerebrovascular disease, even without prior cardiac problems or suspected cardiac cause. Similarities, especially between patients with ischemic stroke and transient ischemic attack, were observed. Our data suggest that all patients with acute cerebrovascular events should receive thorough workup searching for cardiac manifestations.

**Nonstandard Abbreviations and Acronyms**
CEVD: cerebrovascular disease
HS: hemorrhagic stroke
IS: ischemic stroke
SSR: Swiss Stroke Registry
TOAST: Trial of Org 10 172 in Acute Stroke Treatment
VES: ventricular extrasystole

**Clinical Perspective**

**What Is New?**

- Arrhythmia/electrocardiographic changes were the most common cardiac manifestation, followed by myocardial alterations, in all cerebrovascular disease entities, with patients with transient ischemic attack and patients with ischemic stroke showing similar individual cardiac findings.

**What Are the Clinical Implications?**

- All cerebrovascular disease entities should receive a thorough cardiological diagnostic workup in a routine manner, not only to identify potential cardioembolic causes of stroke, but also to prevent cardiac complications.
- Because arrhythmogenic/electrocardiographic and myocardial findings were most common, routine checkups after cerebrovascular disease should comprise Holter electrocardiographic as well as echocardiographic examinations.

The brain and heart are tightly interconnected. Over the past years, knowledge about the interplay between the brain and heart has increased, adding to the growing field of neurocardiology.^1^, ^2^ Cardiac diseases may cause cerebrovascular disease, such as cardiac embolism, resulting in ischemic stroke (heart–brain axis).^3^ On the other hand, findings like cardiac arrhythmia^4^ or damaged myocardial fibers^5^ have frequently been observed to appear after acute cerebral lesions (brain–heart axis).^6^ Increasing evidence suggests that stroke does not only coincide with cardiac pathology due to common risk factors, but instead may provoke cardiac dysfunction.^7^, ^8^, ^9^ One suggested pathway is autonomic nervous system impairment leading to cardiac damage.^9^, ^10^

Different damage mechanisms could lead to disease‐specific patterns of cardiac findings after stroke.^9^ However, most studies in this field either focused on single neurovascular disease entities^11^, ^12^ or included patients with preexisting cardiac conditions.^13^, ^14^, ^15^ Our goal was to determine the fraction and type of cardiac findings occurring in patients within the year after the following 3 cerebrovascular disease (CEVD) types: ischemic stroke (IS), transient ischemic attack (TIA), and hemorrhagic stroke (HS). Aiming for cardiac findings potentially caused by cerebrovascular disease, patients with known cardiac comorbidities or with suspected cardiac cause of a cerebrovascular event were excluded.

## Methods

### Data Availability

Data and analytic material supporting the results of this study can be made available upon reasonable request.

### Ethics

This study was approved by the local ethics commission of the canton of Zurich (Kantonale Ethikkommission Zürich; KEK‐ZH‐Nr. 2014‐0304). Data from patients were included only if general consent for use of data for research purposes was given.

### Study Settings

This retrospective and descriptive study included patients with stroke (IS, TIA, or HS) from the SSR (Swiss Stroke Registry) admitted to the Neurology Department at the University Hospital Zurich between the December 30, 2013 and December 29, 2020. The Strengthening the Reporting of Observational Studies in Epidemiology checklist was used as a reporting guideline.^16^

### Participants

**Figure 1:**
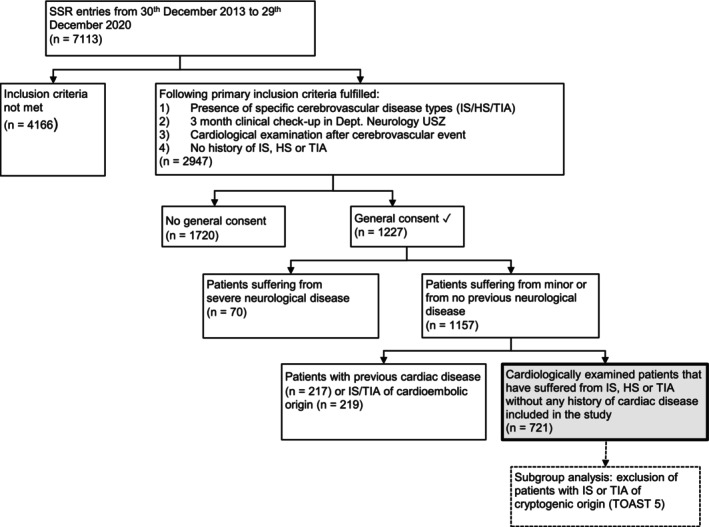
Flowchart showing the exclusion of patients in this investigation. HS indicates hemorrhagic stroke; IS, ischemic stroke; SSR, Swiss Stroke Registry; TIA, transient ischemic attack; TOAST, Trial of Org 10 172 in Acute Stroke Treatment; and USZ, University Hospital Zurich.

Data from patients within the SSR were screened for 3 criteria: (1) presence of TIA, IS, or HS, which had to be a first‐in‐a‐lifetime event; (2) record of neurological checkup at the Department of Neurology within 3 months after CEVD; and (3) 1 cardiological checkup within 1 year after CEVD onset were mandatory. To detect cardiac findings that are truly new and not a consequence of previous disease, we excluded patients with confirmed cardioembolic IS or TIA, known as TOAST 2 (Trial of Org 10172 in Acute Stroke Treatment subtype 2),^17^ as well as patients with preexisting major cardiological or severe neurological diseases, such as severe traumatic brain injury or glioma/brain metastasis. Patients suffering only from minor neurological diseases, which were believed to not affect the brain–heart axis (eg, migraine), were included. Detailed information about the specific previous cardiological and neurological diseases that led to patient exclusion may be found in Table S1 and Table S2. A patient flowchart is depicted in Figure 1.

In a subanalysis, we further excluded patients with IS/TIA due to unknown/multiple cause (TOAST 5), as those potentially include cardioembolic cases.^17^

### Source of Data

Patients fulfilling the criteria mentioned above were eligible for further investigation. All cardiological, neurological, and intensive care reports, created within 1 year after cerebrovascular event, were retrieved from the hospital's clinical information system. Echocardiographic reports as well as cardiovascular magnetic resonance imaging, Coronary Computed Tomography Angiography, or myocardial perfusion single‐photon emission computed tomography were screened, allowing accurate detection of myocardial findings. Clinical and outcome parameters (National Institutes of Health Stroke Scale on admission, as well as National Institutes of Health Stroke Scale and modified Rankin Scale after 3 months) were collected.

### Cardiac Parameters

All medical reports were screened for new cardiac findings/diseases by a medical professional who had access to all data. Ambulatory cardiology reports, echocardiography, long‐term electrocardiography, and advanced cardiac imaging reports were scanned for new cardiac findings. Most cardiac findings were detected in reports from ambulatory cardiac examination (usually performed 3 months after CEVD onset) or from cardiac consultation reports created during hospitalization. To determine whether a finding was truly new, medical admission reports of the CEVD‐related hospital stay were verified. A cardiac parameter finding was considered new if present in a medical report created within 1 year after CEVD, and never mentioned previously in the admission letter. Individual findings of said subgroups were analyzed and compared. Due to the large number of those individual observations, only cardiac findings observed in ≥5% of patients in at least 1 CEVD subgroup were summarized in the tables shown below. Structural findings that could have been present before the index CEVD event (eg, persistent foramen ovale), were not considered. Subsequently, individual cardiac findings retrieved were categorized according to their anatomical and functional origin. In total, 4 cardiac parameter categories were compared, each representing a specific functional entity of the heart: arrhythmogenic findings/electrocardiographic changes, myocardial alterations, valve disease, and findings of insufficient coronary perfusion. Atrial tachycardia was defined as such when atrial rhythm with a frequency >100 bpm was observed, according to latest European Society of Cardiology guidelines.^18^ Structural (eg, atrial enlargement) or functional (eg, heart failure) abnormalities of the myocardium were considered as myocardial alterations. Diastolic dysfunction was diagnosed using official American Society of Echocardiography and the European Association of Cardiovascular Imaging guidelines.^19^ Wall motion abnormalities (including left and right ventricular akinesia/hypokinesia), as well as other atrial/ventricular enlargements or hypertrophic changes, were diagnosed using current recommendations on chamber quantification according to the American Society of Echocardiography and the European Association of Cardiovascular Imaging.^20^ Cardiomegaly was diagnosed using any kind of cardiac imaging method. Chronic and acute heart failure were defined as such, only when fulfilling the necessary criteria according to the European Society of Cardiology.^21^ Findings of insufficient coronary perfusion comprised findings ranging from general coronary heart disease to specific observations, such as severe stenosis of the left marginal artery. Coronary perfusion insufficiencies were diagnosed using advanced cardiac imaging methods (cardiovascular magnetic resonance imaging, coronary computed tomography angiography, or single‐photon emission computed tomography), invasive diagnostics, or ergometry. Electrocardiography findings indicating myocardial ischemia/infarction were only included when interpreted as such by an experienced clinician. Not only moderate or severe, but also mild valve disease was considered significant in this investigation, however only when fulfilling the necessary criteria created by the American Society of Echocardiography in collaboration with the Society for Cardiovascular Magnetic Resonance.^22^

### Statistical Analysis

Descriptive statistics and statistical testing were done using R (version 4.1.2).^23^ Categorical variables are presented as frequencies and percentages, and continuous or discrete variables as median and interquartile ranges (IQRs). To determine whether the frequencies of categorical variables per cerebrovascular subgroups were significantly different, the Pearson χ^2^ test was used. If the values of ≥20% of all cells in the χ^2^ test contingency table were <5, the Fisher exact test was applied to maintain statistical accuracy.^24^ To evaluate how permanent (versus transient) brain ischemia was associated with cardiac findings, patients with IS were directly compared with patients with TIA by applying the Pearson χ^2^ test (or Fisher exact test whenever applicable), as well as by calculating unadjusted odds ratios. Due to limited power and lack of pathophysiological similarities between HS and IS/TIA, no direct comparison of said subgroups was performed. When analyzing the differences of continuous or discrete variables in cerebrovascular subgroups (categorical variables), the nonparametric Kruskal‐Wallis test was applied. *P* values ≤0.05 were considered statistically significant. Bonferroni correction was applied due to multiple testing.

## Results

### Patient Characteristics

Of the 7113 patients screened, 2947 patients were selected according to the primary inclusion criteria described above (Figure 1). Overall, 96.1% of patients with IS, 87.2% of patients with TIA, and 37.2% of patients with HS underwent cardiological examination (*P*<0.001). Seven hundred twenty‐one patients met all inclusion criteria. These patients were divided into 3 subgroups, according to their CEVD (580 IS, 98 TIA, and 43 HS). Patients' characteristics are displayed in Table 1. Patients with TIA were older than patients with IS and HS (*P*=0.156). There were more men than women in the IS subgroup (61.2% men, n=355), whereas more women suffered from HS (58.2%, n=28). Although hyperlipidemia was the most common cardiovascular risk factor in patients with TIA and IS, patients with HS most commonly presented with arterial hypertension. Newly diagnosed (ie, within the 1‐year observation period) hypertensive or hypotensive blood pressure abnormalities were found in 48.8% (n=21) of patients with HS, and observed in 17.2% (n=100) of patients with IS and 16.3% (n=16) of patients with TIA (*P*<0.001). Overall, 10.9% (n=63) of patients with IS, 11.2% (n=11) of patients with TIA, and 23.3% (n=10) of patients with HS stroke were diagnosed with arterial hypertension within the observation period following disease onset (*P*=1). Patients with HS were most severely affected, with a median National Institutes of Health Stroke Scale of 8 (IQR:  3‐13) on admission and 2 (IQR: 0‐5) after 3 months, (*P*<0.001). The median modified Rankin Scale score after 3 months was highest in the HS subgroup (2; IQR, 1‐3), followed by the IS (1; IQR: 0‐2) and the TIA (0; IQR: 0‐0) subgroups (*P*<0.001). Detailed information about CEVD cause can be found in Table 1.

**Table 1:** Baseline, Clinical, and Outcome Characteristics

Variable	Acute ischemic stroke (n=580)	Transient ischemic attack (n=98)	Hemorrhagic stroke (n=43)	*P* value
Baseline characteristics				
Women, n (%)	225 (38.8)	44 (44.9)	28 (58.2)	0.36
Age, y, median (IQR)	64 (53‐73)	70 (61.25‐74.75)	61 (48‐78.5)	0.156*
Clinical characteristics				
Previous neurological disease, n (%)	107 (18.4)	25 (25.5)	12 (27.9)	1.0
History of diabetes, n (%)	71 (12.2)	12 (12.3)	5 (11.6)	1.0
History of arterial hypertension, n (%)	177 (30.5)	50 (51.0)	25 (58.1)	<0.001
History of hyperlipidemia, n (%)	329 (56.7)	62 (63.3)	10 (23.3)	<0.001
History of smoking, n (%)	181 (31.2)	23 (23.5)	6 (14.0)	0.276
Outcome characteristics				
NIHSS on admission, median (IQR)	3 (1‐6)	0 (0‐1)	8 (3‐13)	<0.001*
NIHSS after 3 months, median (IQR)	0 (0‐2)	0 (0‐0)	2 (0‐5)	<0.001*
mRS after 3 months, median (IQR)	1 (0‐2)	0 (0‐0)	2 (1‐3)	<0.001*
Death after 3 months, n (%)	10 (1.7)	0 (0)	2 (4.7)	1.0†
Cause of cerebrovascular disease				
Ischemic stroke and transient ischemic attack				
TOAST 1, n (%)	141 (24.3)	13 (13.2)		
TOAST 3, n (%)	82 (14.1)	8 (8.2)		0.003
TOAST 4, n (%)	68 (11.7)	5 (5.1)		
TOAST 5, n (%)	289 (49.8)	72 (73.5)		
Hemorrhagic stroke				
Hypertension, n (%)			25 (58.1)	
Amyloid angiopathy, n (%)			2 (4.7)	
Trauma, n (%)			1 (2.3)	NA
Vascular lesion, n (%)			2 (4.7)	
Anticoagulant/antithrombotic treatment, n (%)			1 (2.3)	
Unknown, n (%)			12 (27.9)	

The values shown are medians with IQRs or n (numbers of observations) with percentages of patients showing the given finding. For categorial variables, the Pearson χ^2^ test was used to determine significant differences between subgroups. IQR indicates interquartile range; mRS, modified Rankin Scale; NA, not available; NIHSS, National Institutes of Health Stroke Scale; and TOAST, Trial of Org 10 172 in Acute Stroke Treatment.

* If >20% of the 2×3 contingency table cells show a value <5, the Fisher exact test was applFisher exact test was applied.

^†^ Nonparametric Kruskal‐Wallis test was applied.

### Cardiac Findings

In total, 130 different individual cardiac findings were identified, with all patients showing at least 1 new finding. Only 22 of 138 were observed in ≥5% of patients in at least 1 CEVD subgroup. Their frequencies per subgroup are summarized in Table 2.

**Table 2:** Frequencies of Individual Cardiac Findings Present in ≥5% of Patients in at Least 1 Subgroup

Variable	Acute IS (n=580)	TIA (n=98)	HS (n=43)	*P* value (all 3 subgroups)	*P* value (IS vs TIA)	Odds ratio (IS vs TIA)
Arrhythmogenic findings/electrocardiofgraphic changes, n (%)						
Supraventricular extrasystole	393 (67.8)	57 (58.2)	7 (16.3)	<0.001	1.0	1.51 (0.95–2.39)
Ventricular extrasystole total	368 (63.4)	46 (46.9)	8 (18.6)	<0.001	0.046	2.06 (1.3–3.2)
Ventricular extrasystole undetermined	172 (29.7)	28 (28.6)	7 (16.3)	1.0	1.0	1.05 (0.64–1.76)
Atrial tachycardia	137 (23.6)	21 (21.4)	3 (7.0)	0.92	1.0	1.13 (0.66–2.01)
Monomorphic ventricular extrasystole	84 (14.5)	8 (8.2)	0 (0)	0.184	1.0	1.9 (0.88–4.71)
Polymorphic ventricular extrasystole	77 (13.3)	6 (6.1)	1 (2.3)	0.414	1.0	2.34 (1–6.78)
Sinus arrhythmia	67 (11.6)	10 (10.2)	0 (0)	1.0	1.0	1.15 (0.56–2.6)
Supraventricular tachycardia	62 (10.7)	11 (11.2)	1 (2.3)	1.0	1.0	0.95 (0.47–2.07)
Bradycardia	48 (8.3)	12 (12.2)	1 (2.3)	1.0	1.0	0.65 (0.32–1.39)
Ventricular bigeminal rhythm	34 (5.9)	4 (4.1)	0 (0)	1.0*	1.0*	1.46 (0.5–5.8)
Nonsustained ventricular tachycardia	26 (4.5)	9 (9.2)	0 (0)	0.966	1.0	0.46 (0.2–1.17)
QTc prolongation	14 (2.4)	2 (2.0)	4 (9.3)	0.598*	1.0*	1.19 (0.27–10.93)
Atrioventricular block, I–III	34 (5.9)	5 (5.1)	2 (4.7)	1.0	1.0	1.16 (0.43–3.89)
Myocardial alterations, n (%)						
Diastolic dysfunction	153 (26.4)	23 (23.5)	10 (23.3)	1.0	1.0	1.17 (0.69–2.03)
Left atrial enlargement	108 (18.6)	22 (22.4)	6 (14.0)	1.0	1.0	0.79 (0.46–1.4)
Septal hypertrophy	60 (10.3)	8 (8.2)	0 (0)	1.0	1.0	1.3 (0.59–3.25)
Hypokinesia	37 (6.4)	6 (6.1)	3 (7.0)	1.0	1.0	1.04 (0.42–3.11)
Right atrial enlargement	23 (4.0)	7 (7.1)	1 (2.3)	1.0	1.0	0.54 (0.22–1.53)
Left ventricular heart failure	27 (4.7)	2 (2.0)	3 (7.0)	1.0*	1.0*	2.34 (0.57–20.64)
Cardiomegaly	15 (2.6)	3 (3.1)	3 (7.0)	1.0*	1.0*	0.84 (0.23–4.62)
Concentric hypertrophy	14 (2.4)	0 (0.0)	3 (7.0)	0.966*	1.0*	NA
Valve disease findings, n (%)						
Mitral valve insufficiency	64 (11.0)	13 (13.3)	2 (4.7)	1.0	1.0	0.81 (0.42–1.68)
Aortic valve insufficiency	35 (6.0)	7 (7.1)	3 (7.0)	1.0	1.0	0.84 (0.35–2.3)

The Pearson χ^2^ test was used to identify significant differences between subgroups. The number of observations (n) in each cerebrovascular disease subgroup is displayed, including the *P* value and odds ratio with its 95% CI. HS indicates hemorrhagic stroke; IS, ischemic stroke; NA, not available; and TIA, transient ischemic attack.

* If >20% of the 2×3 contingency table cells show a value <5, the Fisher exact test was applied.

Arrhythmogenic findings/electrocardiographic changes were the most common cardiac abnormality in all subgroups: 81.7% of patients with IS (n=474), 71.4% of patients with TIA (n=70), and 55.8% of patients with HS (n=24) developed at least 1 type of electrocardiographic change within 1 year after CEVD onset (*P*<0.001). These comprised monomorphic and polymorphic ventricular extrasystoles (VES) in 14.5% (n=84) and 13.3% (n=77) of patients with IS, in 8.2% (n=8) and 6.1% (n=6) of patients with TIA, and in 0% and 2.3% (n=1) of patients with HS. The frequency of VES in general was significantly different between subgroups (*P*<0.001). Supraventricular extrasystoles were even more common: 67.8% (n=393) in patients with IS, 58.2% (n=57) in patients with TIA, and 16.3% (n=7) in patients with HS (*P*<0.001). Atrial tachycardia was common as well; 23.6% (n=137) of patients with IS, 21.4% (n=21) of patients with TIA, and 7% (n=3) of patients with HS experienced atrial tachycardia (*P*=0.92). A detailed description of electrocardiographic finding frequencies are shown in Table 2. The only electrocardiographic change most common in patients with HS were QTc‐prolongations, found in 9.3% (n=4) of patients with HS, in 2.4% (n=14) of patients with IS, and 2% (n=2) of patients with TIA (*P*=0.598).

**Figure 2:**
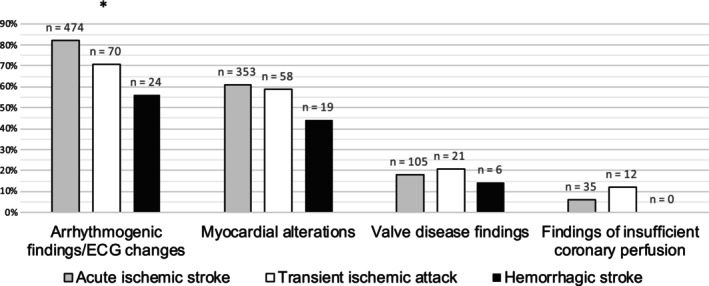
Proportion of cardiac findings observed, categorized according to their anatomical and functional region of origin. Cardiac finding categories showing significant differences in proportions between the CEVD subgroups are marked using an asterisk (*). The Pearson χ^2^ test was applied. CEVD indicates cerebrovascular disease.

Myocardial alterations occurred in 60.9% (n=353) of patients with IS, 59.2% (n=58) of patients with TIA, and 44.2% (n=19) of patients with HS (*P*=0.396, Figure 2). Considering wall motion abnormalities, fewer patients were observed to have akinesia than hypokinesia; 7.5% (n=44) of patients with IS, 7.1% (n=7) of patients with TIA, and 9.3% (n=4) of patients with HS showed at least 1 form of wall motion abnormality (*P*=1). Overall, 12.4% (n=72) of patients with IS, 8.2% (n=8) of patients with TIA, and 7% (n=3) of patients with HS either showed septal or concentric hypertrophy (*P*=1). Overall, 87.6% (n=63) of patients with IS, 87.5% (n=7) of patients with TIA, and 100% (n=8) of patients with HS showing signs of septal or concentric hypertrophy were simultaneously either previously or newly diagnosed with arterial hypertension. Meanwhile, cardiomegaly coincided with arterial hypertension in 60% (n=9) of patients with IS and 100% (n=3) of patients with HS (*P*=0.51). Takotsubo cardiomyopathy was found in 2 patients with HS (4.6%); however, only 1 was confirmed using invasive diagnostics (2.3%). Two patients with IS (0.3%) were diagnosed with Takotsubo cardiomyopathy, whereas in 6 patients, (1%) the diagnosis was suspected but not confirmed. No patients with TIA showed any signs of Takotsubo cardiomyopathy.

Valve disease findings were similarly distributed between subgroups: 18.1% (n=105) of patients with IS, 21.4% (n=21) of patients with TIA, and 14% (n=6) of patients with HS (*P*=1). The 2 most common findings were aortic and mitral valve insufficiency (Table 2).

Insufficient coronary perfusion findings were least prevalent in all 3 subgroups. The proportions of findings indicating insufficient coronary perfusion in the 3 groups were not significantly different (6.0% [n=35] of patients with IS, 12.2% [n=12] of patients with TIA, and 0% of patients with HS; *P*=0.076). In 1.4% (n=8) of patients with IS, signs of myocardial infarction within the year following brain ischemia were observed, whereas none of the other patients showed said complication. Common individual signs of insufficient coronary perfusion in the IS subgroup were exercise‐induced myocardial ischemia (1.2%, n=7), severe coronary sclerosis (1.6%, n=9), and myocardial infarct scars (1.6%, n=9). Patients with TIA were diagnosed with exercise‐induced ischemia (2%, n=2), undetermined coronary sclerosis, and severe coronary sclerosis (3.1% each, n=3). Overall, 7.1% (n=7) of patients with TIA were diagnosed with some type of coronary artery sclerosis (by coronary computed tomography angiography or interventional coronary angiography), whereas only 3.6% (n=21) of patients with IS showed said finding (*P*=1).

### TIA Versus Acute IS

Overall, patients with IS and TIA showed similar frequencies of cardiac findings (Table 3). When directly comparing IS with TIA, none of 4 cardiac parameter categories showed significantly different frequencies (Figure 2). Only the total amount of VES differs significantly between those 2 patient groups (*P*=0.046). Polymorphic VES and left ventricular heart failure both occurred 2.34 times more often in patients with IS than in patients with TIA (*P*=1). Overall, 7.1% of patients with TIA developed coronary stenosis (n=7), whereas only 3.6% of patients with IS were diagnosed with this finding (using coronary computed tomography angiography or interventional coronary angiography; *P*=1).

**Table 3:** Frequencies of the Most Common Individual Cardiac Findings

Variable	Acute ischemic stroke (n=580)		Transient ischemic attack (n=98)		Hemorrhagic stroke (n=43)	
Supraventricular extrasystole, n (%)	1	393 (67.8)	1	57 (58.2)	3	7 (16.3)
Ventricular extrasystole total, n (%)	2	368 (63.4)	2	46 (46.9)	2	8 (18.6)
Diastolic dysfunction, n (%)	3	153 (26.4)	3	23 (23.5)	1	10 (23.3)
Atrial tachycardia, n (%)	4	137 (23.6)		21 (21.4)		3 (7)
Left atrial enlargement, n (%)		108 (18.6)	4	22 (22.4)	4	6 (14)

The 4 most common individual cardiac findings in each subgroup are shown, ranked to their proportion of occurrence.

### Subgroup Analysis Excluding Cryptogenic IS/TIA

Overall, 49.8% (n=289) of cases with IS and 73.5% (n=72) of cases with TIA were of cryptogenic origin (*P*<0.001, Table 1). Electrocardiographic changes remained significantly more common in patients with IS (*P*=0.001). However, when comparing TIA/IS directly, significant differences in cardiac parameter groups disappeared. Only supraventricular extrasystoles (*P*<0.001) remained more common after IS and TIA than HS.

## Discussion

Our study shows that patients without known preexisting cardiac abnormalities, suffering from IS, TIA, or HS of noncardioembolic origin frequently show similar cardiac findings in the year following CEVD. The majority of cardiac findings were changes in electrocardiography or functional/structural alterations of the myocardium.

Arrhythmogenic/electrocardiographic findings were most frequent, being significantly more common in patients with TIA and IS than in patients with HS. This observation is important, because cardiac arrhythmia is a frequent cause of death after stroke.^5^, ^9^, ^15^ Our study detected more electrocardiographic changes after stroke than others (potentially due to consideration of long‐term [1 year] follow‐up data).^25^ In our investigation, monomorphic and polymorphic VES were most frequent in patients with IS, followed by patients with TIA. This high prevalence (especially polymorphic ventricular extrasystole occurring 2.34 more often in patients with IS than in patients with TIA) should be kept in mind during a diagnostic workup, because frequent VES, in the context of structural heart disease, may increase the probability of sudden cardiac death,^26^ and has been associated with increased mortality and higher hospitalization rates, even in patients with structurally normal hearts.^27^, ^28^ However, ventricular and supraventricular extrasystoles are known to be frequent in healthy individuals as well (up to 40%–75% of healthy individuals show VES in a 24‐ to 48‐hour Holter electrocardiography^29^), hence limiting the clinical significance of this finding.^30^ Surprisingly, nonsustained ventricular tachycardia was most common in patients with TIA (9.2% compared with 4.5% in patients with IS). An increasing incidence proportion of ventricular arrhythmia within 30 and 90 days after TIA (1.2% to 2.1%) has already been reported.^31^ Our investigation seems to show a further increase of ventricular arrhythmia in the following months, even in previously cardiologically healthy and noncardioembolic patients with TIA. QTc‐prolongation was the only electrocardiographic finding most common in patients with HS, indicating that this subgroup is at particular risk of developing severe cardiac arrhythmia (ie, torsade de pointes).^32^ Another research group found similar frequencies of QTc prolongations in patients with HS.^6^, ^33^ However, the observation that QTc prolongations were just as common in patients with TIA as in patients with IS is new.

The most common myocardial finding was diastolic dysfunction, found in >20% of patients in all subgroups. The average proportion of diastolic dysfunction in the general population aged >65 years was previously estimated between 3.1% and 5.5%.^34^ Although diastolic dysfunction is known to occur frequently after IS and HS,^9^, ^35^ this investigation showed that patients with TIA are equally prone to diastolic dysfunction. Diastolic dysfunction was proven to be independently associated with functional dependence and death up to 1 year after ischemic stroke^14^ and therefore has to be taken seriously. Left atrial enlargement was observed in ≥14% of all patients in every subgroup, being especially common in patients with TIA. Due to its association with congestive heart failure,^36^ atrial fibrillation,^37^ and recurrent stroke,^37^ clinicians should incorporate this finding into prognosis estimation. As expected, manifestations of long‐standing arterial hypertension, cardiomegaly, septal/concentric hypertrophy, left ventricular heart failure, and myocardial hypokinesia were more frequently observed in patients with HS than patients with IS and TIA (though not significantly). Interestingly, all cardiomegaly and hypertrophic findings within the HS subgroup could be attributed to either newly or previously diagnosed arterial hypertension. In the IS subgroup, however, only 87.6% of septal/concentric hypertrophy and 60% of cardiomegaly cases coincided with arterial hypertension, indicating that other (either preexisting or recently developed) factors may contribute to structural myocardial changes after ischemic stroke. Cardiomegaly is an independent predictor of death after stroke.^38^ Wall motion abnormalities were observed in >7% of all patients in every subgroup. Left ventricular systolic dysfunction rates of 17.3% within the first 24 hours after stroke onset have been described.^39^ On the other hand, similar heart failure incidence rates (6.4%) within a 5‐year observation period after stroke onset has been reported,^40^ whereas 4.7% of our patients with IS developed left ventricular heart failure within 1 year after CEVD onset. This indicates that a significant part of left ventricular dysfunction directly after stroke might be transient but with a significant number of permanent heart failure cases, most of which developing within the first 12 months after IS onset. Compared with patients with TIA, patients with IS are 2.34 times more prone to developing left ventricular heart failure. Although the percentage of left ventricular heart failure after TIA matches previous observations, the finding that noncardioembolic patients with TIA develop wall motion abnormalities to a similar extent as patients with IS may have been underestimated to date.^41^ The percentage of suspected or confirmed patients with Takotsubo cardiomyopathy (4.6%) in the HS subgroup was similar to previous observations in patients with subarachnoid hemorrhage.^42^ Although 1.3% of our patients with IS showed Takotsubo cardiomyopathy signs, another investigation showed this diagnosis to a similar extend (1.2% of patients with IS).^43^

Compared with other cardiac categories, signs of insufficient coronary perfusion were not common in patients with TIA and IS and virtually nonexistent in patients with HS. Patients with TIA showed almost twice as many findings indicating coronary perfusion insufficiency than patients with IS. This difference decreased when excluding cryptogenic cases of IS/TIA, significantly reducing sample size/statistical power. Insufficient coronary perfusion findings in patients with TIA mainly consisted of coronary stenosis. Patients with TIA were older (although not significantly) and more at risk for developing coronary disease.^44^ Our study confirms the risk of developing myocardial infarction after stroke, with 1.4% of patients with IS showing findings indicative of myocardial infarction.^45^ Other investigations showed even higher incidence rates (11.1%) of acute coronary syndrome after IS in a 5‐year follow‐up study, potentially indicating further atherosclerotic disease progression (emphasizing the importance of secondary preventive measures).^40^ Interestingly, no patients with TIA and HS showed signs of myocardial infarction. However, this observation must be interpreted with caution, because those 2 subgroups consisted of significantly fewer patients. Summarizing the above, signs of coronary perfusion insufficiency in previously cardiologically healthy patients with IS consisted of myocardial infarction and coronary stenosis. In the TIA subgroup, these findings mainly consisted of coronary stenosis, which was frequently compared with IS/HS.

### Strengths and Limitations

The large number of included patients is a strength of this study. Moreover, rigorously excluding patients with previous cardiac or neurological diseases, as well as cardioembolic stroke cases, increased the accuracy of detecting new cardiac findings. To depict cardiac findings as accurately as possible, a large and diverse number of medical reports were screened.

One limitation is that this investigation is a single‐center study. Another one being the different subgroup sizes, particularly the TIA and ICH groups, rendered statistical comparisons more difficult. The problem of differentiating between preexisting cardiac conditions and newly developed cardiac disease, as well as providing a direct causal link between the conditions, is difficult in clinical practice, and therefore represents an important limitation of this article.^6^ Furthermore, the admission letter might lack information about subclinical cardiac findings, wrongly interpreting them as new in the cardiac examination following the CEVD. However, we tried to exclude patients with preexisting cardiac conditions as rigorously as possible to study the frequency and type of new cardiac findings after CEVD. At the same time, this approach led to a selection of patients with stroke of noncardiac cause, thus emphasizing patients with stroke of atherosclerotic, microvascular, or other origin. Additionally, the types of examination performed during cardiological checkups may vary significantly, because some underwent advanced/invasive diagnostic steps, whereas others received only minor diagnostics. Although almost all patients with TIA/IS received cardiac checkups, only 37.2% of patients with HS received said examination. Thus, selection bias is possible. As another limitation, the long follow‐up observation period can be considered, because certain cardiac findings observed might not be stroke‐related but consequences of other independent diseases or because of simple atherosclerotic progression.

## Conclusions

New cardiac findings after noncardioembolic IS, TIA, and HS in patients without preexisting cardiac disease are frequent and similar, but with arrhythmogenic/electrocariography findings differing significantly between subgroups. Patients with IS usually have cardiac follow‐up, which is not the case for patients with TIA/HS (96.1% versus 87.2% and 37.2%). Patients with TIA and IS showed a similar pattern of cardiac finding frequencies after disease onset (Table 3). Although hypertensive myocardial disease in patients with HS was expected, electrocardiographic changes (eg, QTc prolongation) were frequently observed as well. This, and the fact that patients with TIA develop similar cardiac findings as patients with IS after disease onset, highlights the importance of thorough cardiac workup in all 3 CEVD types, not only to identify potential cardioembolic causes of stoke, but also to prevent serious cardiac complications or even death. Cardiac surveillance should comprise echocardiographic and Holter electrocardiographic examinations, because electrocardiographic and myocardial changes were the most common manifestations. However, further research on this topic is needed, including a prospective study design ensuring that all CEVDs have received the same cardiac checkup to minimize bias. Furthermore, investigation of stroke‐related characteristics (eg, localization or size) leading to specific cardiac manifestations is warranted.

## Sources of Funding

The project was supported by the Baugarten Foundation, the Swiss National Science Foundation (310030_200703 and PP00P3_170683), and the University Hospital of Zurich Clinical Research Priority Program Stroke.

## Disclosures

None.

## Supporting information

Data S1
