# Supplementary material for: Cardiac Findings Following Cerebrovascular Disease
Source: J Am Heart Assoc. 2024 Aug 27;13(17):e034131. doi: 10.1161/JAHA.124.034131 (PMC11646524; doi:10.1161/JAHA.124.034131)
Supplement: Supplementary file 1 — Data S1 [file JAH3-13-e034131-s001.pdf]

SUPPLEMENTAL MATERIAL

Table S1. Summary of previously diagnosed cardiological diseases that led to patient exclusion.

Preexisting major cardiological diseases	Ischemic stroke	Transient ischemic attack	Hemorrhagic stroke
Acute coronary syndrome	1	0	0
Coronary heart disease	16	2	1
ST-elevation myocardial infarction	4	1	0
Non-ST-segment-elevation myocardial infarction	1	0	1
Coronary sclerosis (not further specified)	1	0	2
Aortic valve stenosis	10	1	0
Aortic valve insufficiency	8	1	0
Mitral valve stenosis	2	1	0
Mitral valve insufficiency	9	1	2
Tricuspid regurgitation	2	0	1
Mitral valve repair	4	1	1
Tricuspid valve repair	1	0	0
Aortic valve repair	11	3	2
Mitral valve repair	1	0	3
Congenital heart disease (not further specified)	3	0	0
Ventricular septal defect	3	0	0
Atrial septal defect	2	0	0
Combined heart disease (valvular/hypertensive)	9	1	0
Combined heart disease (coronary/hypertensive/rhythmogenic)	3	0	0
Combined heart disease (rhythmogenic/hypertensive)	4	0	1
Combined heart disease (valvular/coronary)	1	1	0
Combined heart disease (rhythmogenic/valvular)	1	0	1
Rhythmogenic heart disease	2	1	0
Hypertensive heart disease	17	6	1
Valvular heart disease	6	1	1
Concentric hypertrophy	1	0	0
Septal hypertrophy	1	0	1
Atrial tachycardia	1	0	0
Atrial flutter	2	0	0
Non sustained ventricular tachycardia	1	0	0
Atrioventricular nodal reentry tachycardia	1	0	0
Atrial fibrillation (not further specified)	18	1	4
Paroxysmal atrial fibrillation	15	0	2
Atrial fibrillation with tachycardia	16	3	1
Atrial fibrillation (normocardiac)	7	0	2

Atrial fibrillation with bradycardia	3	0	0
Mixed atrial flutter/fibrillation	1	0	0
Bradycardia	2	0	0
Sick Sinus Syndrome	9	0	0
Left bundle branch block	2	1	0
Right bundle branch block	0	1	0
Paroxysmal supraventricular tachycardia	1	0	1
Tachyarrhythmia (not further specified)	1	0	0
Symptomatic ventricular extrasystole	2	0	0
Long QT syndrome	1	0	1
Atrioventricular block grade 1	3	1	0
Atrioventricular block grade 2	6	1	0
Atrioventricular block grade 3	5	1	0
Pacemaker Implantation	5	0	0
Left ventricular heart failure	1	0	1
Systolic and diastolic heart failure	2	0	0
Diastolic heart failure	1	0	0
Hypokinesis	0	0	1
Congestive heart failure	6	0	1
Dilated Cardiomyopathy	1	0	1
Hypertrophic cardiomyopathy	2	0	0
Hypertrophic obstructive cardiomyopathy	2	0	0
Endocarditis	4	1	0
Pericarditis	2	0	0
Myocarditis	1	0	0
Rheumatic fever	2	0	0
Hypertensive emergency	1	1	0
Pulmonary arterial hypertension	3	1	1
Atrial myxoma	1	0	0
Cardiac metastases	1	0	0
Postpericardiotomy syndrome	1	0	0
Cardiomegaly	1	0	0
Loeys-Dietz syndrome (with cardiac manifestations)	1	0	0
Heart transplant	1	0	1
Pericardial tamponade	1	0	0
Percutaneous closure of patent foramen ovale	1	0	0
Thoracic endovascular aortic repair	1	0	0
Catheter ablation	1	0	1
Transposition of the great vessels	0	1	0
**Total**	**264**	**34**	**36**

Table S2. Summary of previously diagnosed severe neurological diseases that led to patient exclusion.

Previous severe neurological diseases	Ischemic stroke	Transient ischemic attack	Hemorrhagic stroke
Arteriovenous malformation	1	1	1
Anaplastic hemangiopericytoma	1	0	0
Brain metastases	3	0	3
Meningioma	14	3	0
Glioblastoma	1	0	2
Glioma	1	1	0
Hemangioma	1	0	0
Oligodendroglioma	1	1	0
Fibrillary astrocytoma	1	0	0
Ependymoma	1	0	0
Intracranial mass (of unclear dignity)	3	0	1
Radiation-induced brain necrosis	0	0	1
Ventriculoperitoneal shunt	0	1	0
Multiple sclerosis	3	0	0
Moderate traumatic brain injury	1	0	0
Severe traumatic brain injury	1	0	0
Epilepsy	22	2	2
**Total**	**55**	**9**	**10**
